# Investigation of Anti-Cancer Properties of Novel Curcuminoids in Leukemic Cells and Dalton Lymphoma Ascites Model

**DOI:** 10.3390/ijms26073186

**Published:** 2025-03-29

**Authors:** Vijayalakshmi Sudarshan, P. Shyamjith, Sujeet Kumar, Febina Ravindran, Bibha Choudhary, Subhas S. Karki

**Affiliations:** 1 Department of Pharmaceutical Chemistry, Dr. Prabhakar B Kore Basic Science Research Centre, Off-Campus, KLE College of Pharmacy (A Constituent Unit of KAHER-Belagavi), Bengaluru 560010, India; viji2587@gmail.com; 2 Institute of Bioinformatics and Applied Biotechnology, Electronic City Phase 1, Bengaluru 560100, India; shyamjithp03@gmail.com (P.S.); feina28@gmail.com (F.R.); 3 Department of Pharmaceutical Chemistry, NITTE College of Pharmaceutical Sciences (A Constituent College of Nitte Deemed to Be University, Mangaluru), Bengaluru, 560064, India; klempharma@gmail.com

**Keywords:** curcuminoids, leukaemia, cyclopentanone derivatives, monoketone, cytotoxicity, apoptosis

## Abstract

Leukemia, one of the major causes of cancer death, ranks 11th worldwide among cancer-related deaths. The current treatment of leukemia faces challenges recently due to a high burden of side effects. It is well established that curcumin has anticancer and tumor-suppressing activities in several cancers in addition to leukemia. Accordingly, 15 derivatives were designed and prepared to improve the shortcomings of curcumin, such as poor aqueous solubility, chemical instability, and low bioavailability. All 15 were evaluated for cytotoxicity against the leukemic cell line MOLT-4, which led to the prioritization and further evaluation of compound curcuminoid (2*E*,5*E*)-2,5-*bis*((3-(4-nitrophenyl)-1-phenyl-1*H*-pyrazol-4-yl)methylene)cyclopentan-1-one **5i**. 5i. Compared to curcumin, 5i was significantly more effective in inducing mitochondrial dysfunction in MOLT-4 cells; hence increased ROS production and cytotoxicity. Treatment groups showed change in mitochondrial membrane potential by flow cytometry analysis. Moreover, tumor volume reduction observed with 5i treatment in Dalton’s Lymphoma model was accompanied with low toxicity. Intrinsic pathways of apoptosis was initiated by compound 5i that lowered Bcl-2 expression while augmenting cytochrome c, Bak and Bax levels both in vivo and in vitro. These results showcase the potent antiproliferative as well as cytotoxic effects of 5i at nanomolar doses against leukemia being at least 60 times more effective than curcumin.

## 1. Introduction

Leukaemia is a type of cancer that involves uncontrollable production and accumulation of blast or immature atypical blood cells in the bone marrow and peripheral blood. It ranks among the top fifteen cancers diagnosed globally [1]. Approximately 3.4% of cancer-related deaths worldwide in 2022 were attributed to leukaemia [2]. Although it can affect individuals at any age, most cases are diagnosed among children and older adults [3]. The cause of leukaemia is not fully established; however, it is believed to be a combination of genetic and environmental factors [4]. New strategies used in cancer treatment include apoptosis induction, gene expression alteration, and signal transduction pathway inhibition. Apoptosis induction allows cancer cells to die selectively without affecting the normal ones. This can be achieved by altering the level of particular proteins responsible for apoptosis regulation, such as Bax, Bcl-2, caspase-3, caspase-9, tubulin, and various kinases. The enhancement of apoptotic cell death is correlated with the upregulation of the pro-apoptotic protein Bax or the downregulation of the anti-apoptotic protein Bcl-2. Caspases that constitute a proteolytic enzyme family foster apoptosis development significantly, and initiator caspases like caspase-9 and effector or apoptotic caspase-3 are strongly imperative to the progression of apoptosis [5].

Exploration and development of drugs from the secondary metabolites obtained from medicinal plants have always been the basis for searching for anticancer agents. Much interest has been drawn in curcumin, a linear diarylheptanoid natural product that contains two oxygen-substituted aryl groups connected by a seven-carbon chain [6]. This phytochemical exhibits a wide range of biological activities, such as antiarthritis, antiatherosclerosis, antibacterial, antidiabetic, antifungal, antihypertensive, antihyperlipidemic, anti-inflammatory, antitumour, antiphlogistic, antipsoriatic, antithrombotic, and antihepatotoxic properties [7]. Curcumin also shows a cytotoxic effect, causes cell death in different leukemic cell lines, and downregulates the WT1 and FLT3 protein expressions related to cell proliferation [1]. Curcumin exerts its anticancer activity by inhibiting key biological pathways such as mutagenesis, oncogene expression, cell cycle regulation, tumour progression, and metastasis [8]. It modulates signalling pathways, enhances tumour suppressor proteins (p53, p21, p27), and promotes apoptosis by reducing pro-survival gene products [9]. Nonetheless, the clinical efficacy of curcumin is, to a small extent, influenced by its water solubility, bioavailability, instability, rapid metabolism, and excretion [10,11]. The limited bioavailability of curcumin arises from its chemical and biological instability when faced with physiological conditions [12].

Several researchers have demonstrated that the modified curcumin analogues have addressed the shortcomings of curcumin by enhancing solubility, bioavailability, and therapeutic efficacy; thus, more bioactivity is observed with reduced toxicity. These changes occur at active sites such as the aromatic side chain, linker chain, diketo functionality, and active methylene group [13,14,15]. Numerous investigations examining the structure–activity relationship of curcumin derivatives/analogues have indicated that transforming the diketone variant into a monoketone variant boosts its stability, pharmacological effectiveness, and solubility. This alteration has gained considerable interest since the 1,3-dicarbonyl structure is highly reactive and promotes degradation through hydrolysis [10]. Several researchers have observed that the analogues of curcumin bearing a cyclopentanone ring showed enhanced anticancer properties and retained their stability for 72 h. In addition, the breakdown of diarylpentadienone was significantly slower and at a lower rate when contrasted with curcumin [16,17].

In addition to changing the diketone version of curcumin into the monoketone variant, it has been noted that replacing the benzene ring with a heteroaromatic ring might preserve or even improve the cytotoxic properties of curcumin’s monoketone derivatives [18]. Heterocyclic compounds containing nitrogen are noteworthy and unique in synthetic and medicinal chemistry. Among these, pyrazoles, which are five-membered heteroaromatic rings, represent a highly significant category of compounds that play a crucial role as key bioactive intermediates in the quest for new therapeutic agents in medicinal research [19,20]. Pyrazole-based structures that hold pharmacological importance exhibit diverse activities, including antiviral, antibacterial, antimalarial, anti-inflammatory, antidiabetic, antiglaucoma, and, most significantly, anticancer effects [21,22,23,24,25,26].

**Figure 1:**
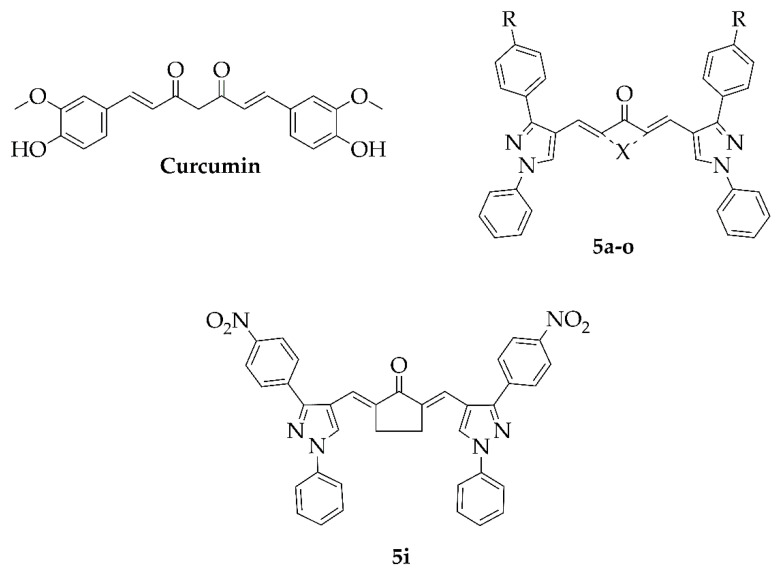
Structures of curcumin, our synthesised curcuminoids, and curcuminoid **5i**.

In this study, 15 curcuminoids were synthesised and characterised by the incorporation of the cyclopentanone, cyclohexanone, and cycloheptanone moiety in the five-carbon linker of curcumin and by the replacement of the aromatic benzene ring with the nitrogen-containing heterocyclic moiety pyrazole. All curcuminoids were screened for in vitro cytotoxicity, and the most active curcuminoid **5i** (2*E*,5*E*)-2,5-*bis*((3-(4-nitrophenyl)-1-phenyl-1*H*-pyrazol-4-yl)methylene)cyclopentan-1-one was further studied experimentally through in vitro and in vivo approaches to unveil the molecular mechanisms underlying its cytotoxic activity (Figure 1).

## 2. Results

### 2.1. Chemistry

#### Synthesis and Characterisation of Curcuminoids

**Scheme 1:**
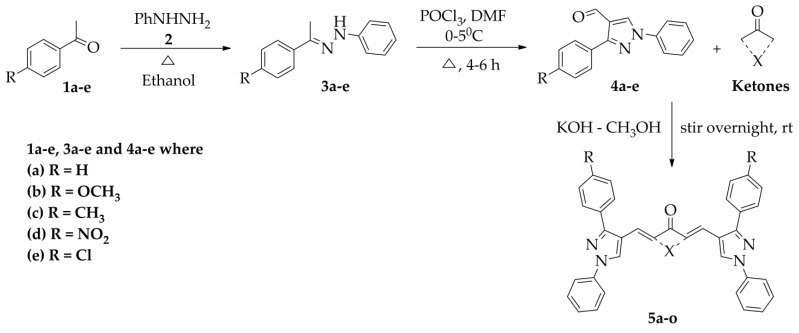
Synthesis of curcuminoids **5a–o**.

A set of fifteen curcuminoids, **5a–o**, were synthesised according to Scheme 1, Table 1. The reaction between 0.05 molar substituted acetophenones (**1a–e**) and 0.05 molar phenyl hydrazine (**2**) in 30 mL of ethanol at reflux for 4 h resulted in the formation of 1-phenyl-2-(1-(4-substituted phenyl)ethylidene)hydrazine (**3a–e**) with yields in the range of 85–95%. The Vilsmeier–Haack reagent was employed to obtain the aldehyde derivatives of 3-(4-substituted phenyl)-1-phenyl-1*H*-pyrazole-4-carbaldehyde (**4a–e**), with yields ranging from 75–85%, by refluxing **3a–e** with phosphorus oxychloride and DMF at low temperatures between 0–5 °C. Lastly, the reactions between different ketones (cyclopentanone, cyclohexanone, and cycloheptanone) (0.001 M) and methanolic potassium hydroxide (0.008 M) with the tetrahydrofuran solutions of 3-(4-substituted phenyl)-1-phenyl-1*H*-pyrazol-4-carbaldehyde (**4a–e**) (0.002 M) produced *bis*((3-(4-substituted phenyl)-1-phenyl-1*H*-pyrazol-4-yl)methylene)ketone analogues (**5a–o**) in yields ranging from 37–86%.

All synthesised curcuminoids **5a–o** were characterised by FTIR, ^1^H/^13^C NMR, and HR-MS spectroscopic data. Stretching absorption bands for C-H aromatic were observed in the range 3082–3009 cm^−1^, C-H aliphatic in 2962–2911 cm^−1^, C=O in 1750–1685 cm^−1^, and C=C aromatic in 1611–1524 cm^−1^ for compounds **5a–o**. Curcuminoid **5i** exhibited characteristic vibration at 1685 cm^−1^ for C=O.

The ^1^H NMR spectra of **5a–o** showed significant signals for pyrazole protons between δ 8.81–7.73 ppm and aromatic protons between δ 7.98–6.98 ppm. The cyclic ketones with different chain lengths showed the presence of -CH_2_- groups between δ 4.2–1.75 ppm. The range for methyl protons in curcuminoids **5c**, **5h**, and **5m** was found to be between δ 2.51–2.35 ppm, while that for methoxy protons in curcuminoids **5b**, **5g**, and **5l** was found to be between δ 3.86–3.85 ppm. For curcuminoid **5i**, prominent signals were observed at δ 3.00 ppm for -CH_2_- groups, at δ 8.18 ppm for pyrazole protons, and between δ 7.80–7.35 ppm for aromatic protons.

The ^13^C NMR spectra of **5a–o** exhibited peaks in the range of δ 207–162 ppm for C=O of ketones, peaks in the range of δ 160–114 ppm for aromatic carbons, peaks in the range of δ 55.45–55.42 ppm for methoxy carbons and at δ 21.59–21.45 ppm for methyl carbons on the benzene ring. Methylene carbon peaks of -(CH_2_)_2_, -(CH_2_)_3_ and-(CH_2_)_4_ of ketones were shown between δ 29.42–21.47 ppm. For curcuminoid **5i**, prominent signals were noted at δ 207.17 ppm for C=O and between δ 153.90–118.14 ppm for aromatic carbons, and at δ 26.57 ppm for methylene carbons in the cyclic ketone -(CH_2_)_2_.

The HR-MS spectrum of curcuminoids **5a–o** showed molecular ion peaks corresponding to their molecular weights; hence, the identity of all the compounds is confirmed. For curcuminoid **5i**, the mass was corroborated by HR-MS with the molecular peak exhibited at 635.2040 (calc. 634.6395).

### 2.2. In Vitro Studies

#### 2.2.1. In Vitro Cytotoxicity Evaluation

All curcuminoids **5a–o** were initially screened at a wide range of concentrations, including 0.1, 1, 10, and 100 μMfor 48 h, with curcumin as the positive control (Table 2). Based on the 3-[4,5-dimethylthiazol-2-yl]-2,5 diphenyl tetrazolium bromide (MTT) assay conducted on MOLT-4 cells, the individual compounds exhibitedhigher cytotoxic activity, except curcuminoids **5b**, **5c**, and **5e**, which showed limited cytotoxicity compared to that of curcumin. From those first-screened compounds, four were selected to be tested at narrow-range concentrations of 20, 40, 60, 80, 100, 200, and 400 nM for 48 h. The most potent was curcuminoid **5i**, which was further analysed.

#### 2.2.2. In Vitro Cytotoxicity Evaluation in Cell Lines

**Figure 2:**
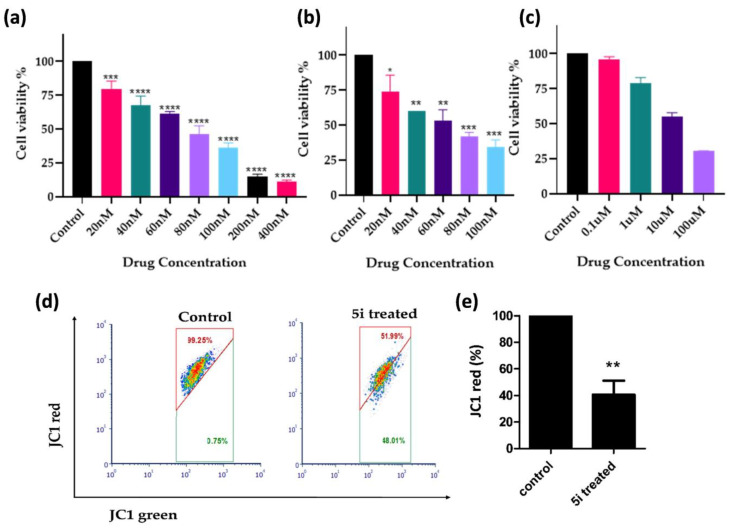
In vitro evaluation of **5i** on cellular processes:(**a**) Cytotoxicity evaluation of **5i** in MOLT-4 cell line using MTT assay. (**b**) Cytotoxicity evaluation of **5i** in MOLT-4 cell line using resazurin assay. (**c**) Cell viability in HEK-293 cells upon the treatment of **5i**. (**d**) Quantification of MMP depicted as a bar graph after 48 h treatment of **5i** on MOLT-4 cells. (**e**) JC-1 staining indicating a change in mitochondrial membrane potential (MMP) upon **5i** treatment. Every experiment was repeated three times and represented as histograms. *p*-value was calculated between control and **5i**-treated groups. *p*-value representation: * (*p*-value ≤ 0.05), ** (*p*-value ≤ 0.01), *** (*p*-value ≤ 0.001), **** (*p*-value ≤ 0.0001).

Based on the broad range data obtained from Section 2.2.1, the cytotoxic activity of curcuminoid **5i** at a narrow range of 20–200nM was performed in MOLT-4 and HEK-293 cells for 48 h by MTT and resazurin assays. Both assays demonstrated an IC_50_below 100 nM (Figure 2a,b). To understand the impact of **5i** on non-cancerous cells, human embryonic kidney cells (HEK293) were treated with increasing concentration of **5i** for 48 h and showed an IC_50_ of 39 μM (Figure 2c), which is a much higher concentration compared to **5i**. These observations demonstrate that **5i** is highly cytotoxic compared to its parent compound curcumin and that **5i** is least cytotoxic in non-cancerous cells.

#### 2.2.3. **5i** Alters Mitochondrial Membrane Potential (MMP; ∆ψm)

The effect of **5i** on the cytotoxicity mechanism was revealed by exposing the MOLT-4 cell line to **5i** for 48 h, then staining with 3-[4,5-dimethylthiazol-2-yl]-2,5 diphenyl tetrazolium bromide (JC-1). In viable cells, JC-1, a cationic dye, accumulates in the mitochondria, forming red fluorescence aggregates. In apoptotic cells, the mitochondrial membrane is leaky to JC-1; hence, no aggregates are formed, resulting in green fluorescence. Figure 2d,e show flow cytometry analysis where there is an increase in the green population upon treatment, with **5i** indicating damage to mitochondria. There was no evidence of mitochondrial damage in the vehicle control.

#### 2.2.4. **5i** Treatment-Induced Apoptotic Activation in MOLT-4 Cells

**Figure 3:**
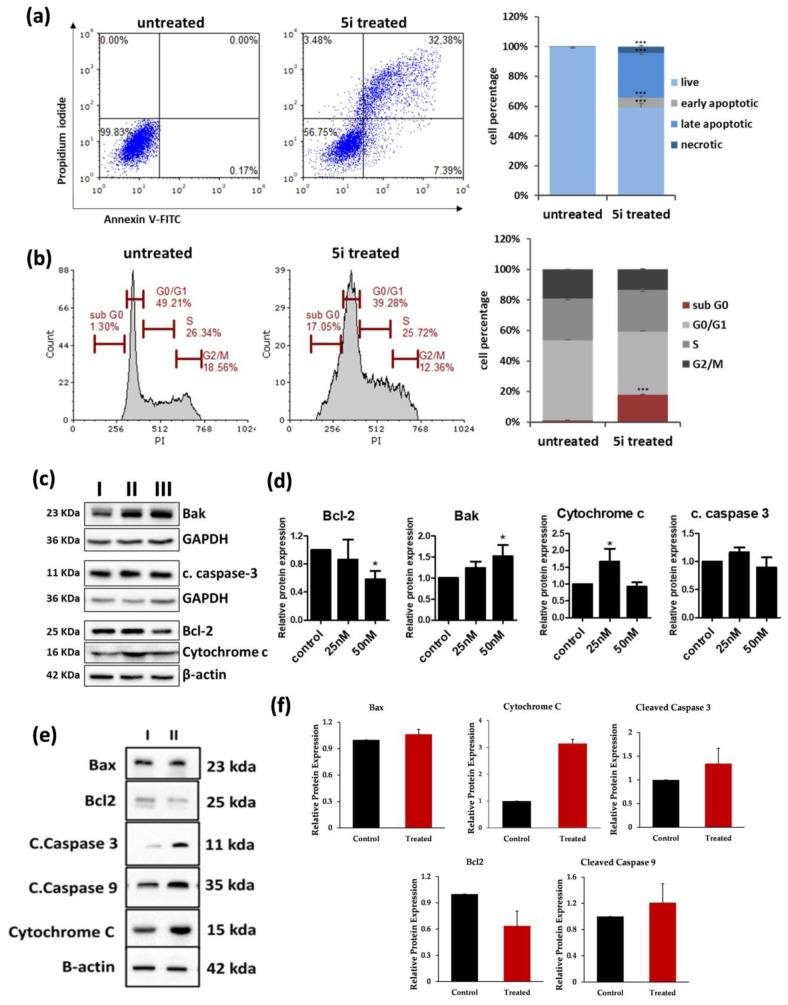
Induction of apoptosis by 5i treatment:(**a**) Histograms and bar graph quantification of cell cycle assay of untreated and **5i**-treated MOLT-4 cells for 48 h. (**b**) Dot plots and bar graph quantifications of apoptosis assay by AnnexinV-FITC/PI double staining of untreated and **5i**-treated MOLT-4 cells for 48 h. (**c**) Western blot images of apoptotic protein markers in untreated and **5i**-treated MOLT-4 cells for 48 h. I, II, and III represent control, 250 nM **5i**, and 500 nM **5i**, respectively. (**d**) Bar blots representing quantification of apoptotic protein markers in **5i**-treated MOLT-4 cells. (**e**) Western blot images of apoptotic protein markers in DLA control and **5i**-treated tumour tissues post 30 days of **5i** treatment at a dosage of 50 mg/kg body weight. I represent untreated tumour and II represent **5i**-treated tumour. (**f**) Quantification of apoptotic protein markers in DLA control and treated tumour. All bar graph data are represented as mean ± SD. All experiments were conducted three times. Significance was plotted based on *p*-value between treated tumour and its paired control condition. *p*-value representation: * (*p*-value ≤ 0.05), *** (*p*-value ≤ 0.001).

To determine the mode of cell death induced by **5i**, an apoptosis assay on **5i**-treated MOLT-4 cells revealed ~6% of cells in the early apoptotic phase, ~30% in the late apoptotic phase, and ~4% in the necrotic phase (Figure 3a). These results indicate that **5i** predominantly induces apoptosis in MOLT-4 cells, with a minimal percentage of necrosis. Additionally, cell cycle analysis of **5i**-treated MOLT-4 cells showed a 17% increase in the apoptotic fraction (sub-G0 phase) and a reduction in the G0/G1 and G2/M phases (Figure 3b). This further supports the conclusion that **5i** treatment primarily triggers apoptosis in MOLT-4 cells.

The **5i**-treated MOLT-4 cells were assessed for protein expression of apoptotic markers using western blotting. Treatment with **5i** resulted in a significant decrease in the anti-apoptotic protein BCL2, alongside a significant upregulation of the pro-apoptotic proteins BAK and *cytochrome c* (Figure 3c,d). Although cleaved caspase 3 was elevated, this change was not statistically significant. Taken together with the disruption of the mitochondrial membrane potential (MMP), flow cytometry analyses, and protein expression data, these findings suggest that **5i** induces the intrinsic apoptotic pathway in MOLT-4 cells.

After testing the drug in an in vitro cell model and deciphering the molecular mechanism of cell death as mitochondria-mediated, we aimed to assess the drug’s impact in vivo. Given that we lack syngeneic mouse models of leukaemia, we opted to test the drug on a mouse lymphoma model. We noted a reduction in tumours in the mouse model described below. We extracted proteins from the tumours of both controls and treated animals and conducted Western blot analysis to examine whether **5i** induced the intrinsic apoptotic pathway. Interestingly, we observed significant changes in the mitochondrial membrane proteins Bcl-2 and Bax (Figure 3e,f) as well as markers of the apoptotic pathway, including cleaved caspase-9, cleaved caspase-3, and cytochrome c in Dalton’s Lymphoma Ascites (DLA) tumours, indicating varied activity of the drug under two different conditions. Further experiments on the drug’s metabolism in the MOLT-4 and DLA models may elucidate the observed differences.

### 2.3. In Vivo Studies

#### **5i** Induced Tumour Regression in DLAMouse Model Without Observable Toxicity

**Figure 4:**
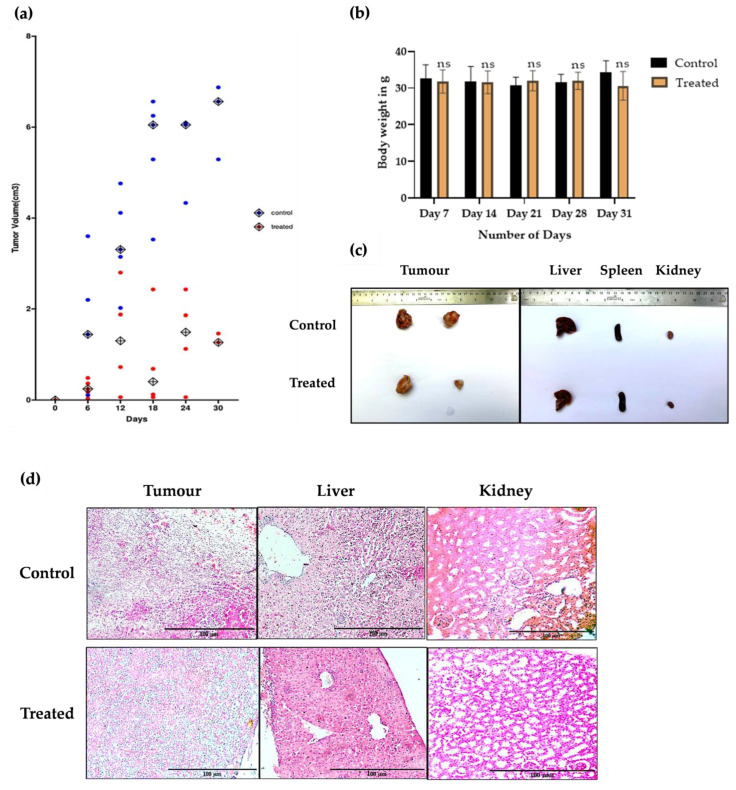
Histological and morphological analysis of the control and 5i-treated DLA mouse model: (**a**) Dot plot representing tumour volume for 30 days of treatment with **5i**. Each dot represents the tumour volume of each individual animal. Diamond represents median tumour volume of untreated or treated for each time point. (**b**) Body weight measurements of control and treated animals (ns represents not significant). (**c**) Tumour, liver, spleen, and kidney of untreated and **5i**-treated animals. (**d**) H&E staining of tumour, liver, and kidney after 30 days of **5i** treatment.

To generate the mouse model, DLA cells were injected into the left thigh region of Swiss albino mice to induce the tumour. After inducing the tumour, the mice were segregated into five control and five treatment groups. The treatment group received 50 mg/kg body weight of **5i** intraperitoneally daily for 31 days, amounting to 30 doses. Throughout the treatment period, tumour volume was measured to assess the effect of **5i** on tumour reduction. Figure 4a demonstrates an increase in tumour volume over 30 days in the control mice. In contrast, the tumour volume did not increase in the presence of **5i**, indicating that **5i** prevented tumour growth. As mentioned in the previous section, this lack of tumour growth can be explained by the induction of mitochondria-mediated cell death in DLA cells.

To assess whether there were any visible signs of drug toxicity, body weight was recorded throughout the experiment. No significant reduction in body weight was observed in the treatment groups (Figure 4b). Furthermore, to determine whether the curcuminoid **5i** affected the liver or kidney—sites of drug metabolism and excretion—the liver, kidney, and spleen were collected, along with the tumour, and their morphology was assessed. As shown in Figure 4c, there was a marked reduction in tumour size. In contrast, no apparent changes were noted between the control and **5i**-treated liver and kidney, suggesting no evident toxicity. The decrease in spleen size was observed alongside a reduction in tumour volume, indicating the clearance of tumour cells by the spleen. This was not observed in the control tumour, where the tumour burden was high, prompting the spleen to enlarge to perform its function. One method of measuring tumour reduction is also to observe the spleen size.

Since no morphological changes were observed to indicate any cellular alterations in the tissues, we prepared sections of the tissues. As shown in Figure 4d, the organisation of the liver and kidney remained intact in both the control and drug-treated samples. The tissue architecture was nearly normal in the **5i**-treated animals, confirming no apparent toxicity. Further experiments using liver and kidney function tests will validate the observations made regarding curcuminoid **5i**.

## 3. Discussion

Curcumin generally acts at cancer’s initiation, promotion, and progression stages through its effects on various genes and proteins. Its antioxidant activity and its reactive oxygen species (ROS) scavenging ability correlate with its anticancer properties, as reactive oxygen species influence several types of cancers. These include leukaemia, lymphoma, melanoma, and sarcomas, as well as genitourinary, breast, ovarian, head and neck, lung, and neurological cancers [27].

Several signalling pathways identified in curcumin’s anticancer effects overlap with the inhibition of NF-κB transcription factor activation. Given that NF-κB-regulated gene products include apoptosis inhibitors (Bcl-2, Bcl-XL, TRAF), regulators of the cell cycle (cyclin D), growth factors (interleukins, VEGF), growth factor receptors, and matrix metalloproteinases, the inhibition of NF-κB trans-activating activity would lead to the downregulation of its targets that govern numerous processes involved in cell proliferation [28].

The activity of curcumin is limited due to its poor solubility in water and low bioavailability. Various strategies are being researched to address these issues, including new natural curcumin analogues derived from turmeric, natural curcumin analogues from other plant species, the production of synthetic curcumin analogues by altering functional groups, reformulating curcumin with various oils, encapsulating curcumin in different amphiphilic copolymers [29], and modifying or synthesizing analogues. These modified structures demonstrate enhanced biological activity compared to standard curcumin, suggesting a promising profile for antitumour activity through the induction of apoptosis and the inhibition of growth and proliferation in various cell lines [10,29].

The current set of curcuminoids was designed by incorporating cyclic ketones within the five-carbon linker analogue of curcumin and by utilizing 3-(4-substituted phenyl)-1-phenyl-1*H*-pyrazole-4-carbaldehyde (**4a–e**) in place of the aromatic benzene ring. This strategy was employed as a curcumin derivative with the cyclic ketone group. It was found to inhibit tumour growth by disrupting cell cycle progression [30], inducing apoptosis [31], and triggering cell cycle arrest in the G2/M phase [32]. These derivatives exhibit stronger antitumour activities than curcumin by activating the ROS-YAP-JNK signalling pathway, inducing mitochondrial dysfunction and apoptosis [33], and inhibiting cell growth through multiple molecular targets, including ROS and ER stress [34]. Furthermore, analogues of curcumin possessing a cyclopentanone ring were discovered to demonstrate cytotoxicity with IC_50_ values below 1 µM and 1.35 µM against MOLT-4, HeLa, PC3, DU145, and KB cancer cell lines [35], as well as against leukaemia K-562 cell lines respectively. Some even displayed effectiveness against leukaemia RPMI-8226 and renal RXF-393 cell lines with GI_50_ values of 0.6 and 0.5 µM, respectively [36].

The present study elucidates the mechanism of cell death through which curcuminoid **5i**(2*E*,5*E*)-2,5-*bis*((3-(4-nitrophenyl)-1-phenyl-1*H*-pyrazol-4-yl)methylene)cyclopent-an-1-one induces potent antitumorigenic activity in vitro against the leukemic cancer cell line and a mouse lymphoma model. A drug can cause cell death via several mechanisms, as previously highlighted [37]. In particular, curcumin derivatives are recognized to induce apoptosis by disrupting the mitochondrial membrane potential and by initiating a cascade of caspase pathways [38,39]. The effect of **5i** was similar and was achieved at a nanomolar concentration, inducing intrinsic apoptotic cell death in MOLT-4 cancer cell lines. This was evidenced by disruption in mitochondrial membrane potential, and the upregulation of pro-apoptotic proteins such as Bax and cytochromec, alongside the downregulation of the anti-apoptotic protein Bcl-2. Following this, it was observed that **5i** induced alterations in Bcl-2-Bax ratios in MOLT-4 cells after treatment, correlating well with the theory that Bax oligomerizes with Bak, forming pores in the mitochondrial membrane that lead to the release of proteins such as cytosolic cytochrome c [40].

Given the encouraging in vitro results, the in vivo activity of **5i** was assessed in the DLA model. Significant regression of tumour growth was observed at a dose of 50 mg/kg with at least 30 doses in the treated groups compared to the control groups. Furthermore, organ damage was minimal due to the efficacy of **5i**.

The H&E staining of control tissue samples revealed more densely packed and stained nuclei than the treated tissue samples. This suggests that **5i** was primarily aimed at proliferating cells. The markers for the intrinsic apoptotic pathway, Bax, cleaved caspase-3, and cleaved caspase-9, were significantly elevated in the tumour samples from treated mice compared to those from untreated controls. These findings, therefore, demonstrate that **5i**, at lower doses, inhibits growth in DLA tumour mouse models and induces cytotoxicity through the intrinsic pathway of apoptosis with minimal side effects.

## 4. Materials and Methods

### 4.1. Chemicals and Instruments

Solvents and reagents were pretested for purity. Silica gel 60 GF_254_ plates from Merck were used to monitor the reaction progress by TLC. The melting point was measured using a DBK melting point apparatus. FTIR spectra were obtained using IR-grade KBr, and the diffuse reflectance technique was employed on the Jasco FTIR 460+. NMR ^1^H/^13^C spectra were recorded between 400 and 500/100 MHz in DMSO-d_6_ and CDCl_3_ on Bruker (Ultraspec AMX 400) and JEOL RESONANCE (JNM-ECZ400S). All chemical shift (δ) values are expressed in ppm with TMS as a reference. The HR-MS spectra were obtained from the Institution of Excellence, Vijnana Bhavan, Mysuru, on the Waters, USA (Xevo G2-XS QTof).

### 4.2. General Procedure for Synthesis of 1-Phenyl-2-(1-(4-substituted phenyl)ethylidene)hydrazine(**3a–e**)

An equal molar solution of 4-substituted acetophenone(**1a–e**) (0.01 M) and phenylhydrazine(**2**) (0.01 M) in ethanol wasplaced in a round bottom flask and refluxed for 4–6h. The solid that separated on cooling was filtered and washed with cold ethanol. The product obtained was immediately used for the next step [41].

#### 4.2.1. (E)-1-Phenyl-2-(1-phenylethylidene)hydrazine **3a**

Yellow-coloured compound. Yield 90%; m.p.96–100 °C (Lit. 102–103 °C [42]).

#### 4.2.2. (E)-1-(1-(4-Methoxyphenyl)ethylidene)-2-phenylhydrazine **3b**

Cream-coloured compound. Yield 91%; m.p.138–140 °C (Lit. 143 °C [43]).

#### 4.2.3. (E)-1-Phenyl-2-(1-(p-tolyl)ethylidene)hydrazine **3c**

Yellow-coloured compound. Yield 85%; m.p.80–84 °C (Lit. 79–80 °C [42]).

#### 4.2.4. (E)-1-(1-(4-Nitrophenyl)ethylidene)-2-phenylhydrazine **3d**

Yellow-coloured compound. Yield 95%; m.p.134–138 °C (Lit. 134.7 °C [44]).

#### 4.2.5. (E)-1-(1-(4-Chlorophenyl)ethylidene)-2-phenylhydrazine **3e**

White-coloured compound. Yield 85%; m.p.100–104 °C (Lit. 102 °C [45]).

### 4.3. General Procedure for the Synthesis of 3-(4-Substituted phenyl)-1-phenyl-1H-pyrazole-4-carbaldehyde (**4a–e**)

Phosphorus oxychloride (0.09 M) was added dropwise through a dropping funnel to previously cooled dimethylformamide (DMF) (0.09 M) at 0–5 °C and stirred until the Vilsmeier complex formed. A solution of 1-phenyl-2-(1-(4-substituted phenyl)ethylidene)hydrazine (**3a–e**) (0.03 M) in DMF was then added dropwise to the reaction mixture. This was stirred at room temperature for a few minutes and refluxed for 6 h. The reaction mixture was subsequently poured into crushed ice and neutralised with sodium bicarbonate to obtain a precipitate. The precipitate was filtered, thoroughly washed with water, dried, and recrystallised using a mixture of chloroform and methanol [46].

#### 4.3.1. 1,3-Diphenyl-1*H*-pyrazole-4-carbaldehyde **4a**

White-coloured compound. Yield 80%; m.p.140–144 °C (Lit. 137–138 °C [46]).

#### 4.3.2. 3-(4-Methoxyphenyl)-1-phenyl-1*H*-pyrazole-4-carbaldehyde **4b**

Off-white-coloured compound. Yield 75%; m.p.100–104 °C (Lit. 100–102 °C [46]).

#### 4.3.3. 1-Phenyl-3-(p-tolyl)-1*H*-pyrazole-4-carbaldehyde **4c**

Cream-coloured compound. Yield 78%; m.p.120–124 °C (Lit. 120–122 °C [46]).

#### 4.3.4. 3-(4-Nitrophenyl)-1-phenyl-1*H*-pyrazole-4-carbaldehyde **4d**

Light-yellow coloured compound. Yield 80%; m.p.160–164 °C (Lit. 163–164 °C [46]).

#### 4.3.5. 3-(4-Chlorophenyl)-1-phenyl-1*H*-pyrazole-4-carbaldehyde **4e**

Buff-coloured compound. Yield 85%; m.p.142–146 °C (Lit. 140 °C [45]).

### 4.4. General Procedure for Synthesis of 2,6-Bis((3-(4-substituted phenyl)-1-phenyl-1H-pyrazol-4-yl)methylene)ketone (**5a–o**)

Potassium hydroxide (0.008 M) in methanol was prepared, after which a batch of tetrahydrofuran containing 3-(4-substituted phenyl)-1-phenyl-1*H*-pyrazole-4-carbaldehyde (**4a–e**) (0.002 M) was added. The mixture was stirred for 20 min under ice-cold conditions. Various ketones (cyclopentanone, cyclohexanone, and cycloheptanone) (0.001 M) were added dropwise. Subsequently, the resulting mixture was stirred at room temperature overnight before being poured into ice-cold water (100 mL). The mixture was neutralised with dilute hydrochloric acid, resulting in a solid that was recrystallised using a mixture of chloroform and methanol.

#### 4.4.1. (2*E*,6*E*)-2,6-*Bis*((1,3-diphenyl-1*H*-pyrazol-4-yl)methylene)cyclohexan-1-one **5a**

Canary yellow coloured, yield 78%, m.p.260–262 °C; IR (KBr) νmax/cm^−1^: 3057, 2939, 1659, 1600, 1503, 1275, 1168, 1059; δ/ppm for ^1^H NMR (400 MHz, DMSO-d_6_): 1.86 (s, 2H, alkyl -CH_2_-), 2.93 (s, 4H, alkyl (-CH_2_-)_2_), 7.36 (t, 2H, Ar, *J* = 16), 7.46–7.61 (m, 16H, Ar), 8.00 (d, 6H, Ar, *J* = 8), 8.82 (s, 2H, Ar); ^13^C NMR (100 MHz, CDCl_3_, δ/ppm): 188.23, 154.85, 139.64, 134.30, 132.37, 129.62, 128.96, 128.81, 128.60, 127.73, 127.53, 127.12, 119.41, 117.42, 28.92, 22.46; HR-MS *m*/*z*: [C_38_H_30_N_4_O]^+^, 559.2488 (calc. 558.6710).

#### 4.4.2. (2*E*,6*E*)-2,6-*Bis*((3-(4-methoxyphenyl)-1-phenyl-1*H*-pyrazol-4-yl)methylene) cyclohexan-1-one **5b**

Light yellow coloured, yield 81%; m.p.212–216 °C; IR (KBr) νmax/cm^−1^: 3049, 2962, 1654, 1596, 1504, 1252, 1177, 1058; ^1^H NMR (400 MHz, CDCl_3_, δ/ppm): 1.96 (p, 2H, alkyl -CH_2_-, *J* = 28), 2.88 (t, 4H, alkyl (-CH_2_-)_2,_ *J* = 12), 3.85 (s, 6H, -CH_3_-), 7.00 (d, 4H, Ar, *J* = 4), 7.32 (t, 2H, Ar, *J* = 16), 7.48 (t, 4H, Ar, *J* = 16), 7.65 (d, 4H, Ar, *J* = 12), 7.79 (d, 4H, Ar, *J* = 12), 7.84 (s, 2H, Ar), 8.12 (s, 2H, Ar); ^13^C NMR (100 MHz, CDCl_3_, δ/ppm): 188.28, 159.98, 154.74, 139.72, 134.08, 130.21, 129.61, 127.90, 127.38, 127.00, 124.92, 119.39, 117.28, 114.27, 55.45, 28.91, 22.45; HR-MS *m*/*z*: [C_40_H_34_N_4_O_3_] ^+^, 619.2710 (calc. 618.7230).

#### 4.4.3. (2*E*,6*E*)-2,6-*Bis*((1-phenyl-3-(p-tolyl)-1*H*-pyrazol-4-yl)methylene)cyclohexan-1-one **5c**

Dark yellow coloured, yield 80%; m.p.240–246 °C; IR (KBr) νmax/cm^−1^: 3018, 2925, 1659, 1598, 1503, 1227, 1172, 1060; ^1^H NMR (400 MHz, DMSO- d_6_, δ/ppm): 1.87 (s, 2H, alkyl -CH_2_-), 2.36 (s, 6H, -CH_3_-), 2.93 (s, 4H, alkyl (-CH_2_-)_2_), 7.31–7.37 (m, 6H, Ar), 7.47–7.54 (m, 10H, Ar), 7.99 (d, 4H, Ar, *J* = 8), 8.80 (s, 2H, Ar); ^13^C NMR (100 MHz, CDCl_3_, δ/ppm): 188.19, 154.95, 139.68, 138.43, 134.14, 129.58, 129.50, 128.84, 127.83, 127.42, 127.00, 119.37, 117.38, 28.91, 22.46, 21.48; HR-MS *m*/*z*: [C_40_H_34_N_4_O] ^+^, 587.2831 (calc. 586.7242).

#### 4.4.4. (2*E*,6*E*)-2,6-*Bis*((3-(4-nitrophenyl)-1-phenyl-1*H*-pyrazol-4-yl)methylene)cyclohexan-1-one **5d**

Dark yellow coloured, yield 40%; m.p.>290 °C; IR (KBr) νmax/cm^−1^: 3069, 2953, 1662, 1597, 1537, 1341, 1269, 1162, 1067; ^1^H NMR (400 MHz, CDCl_3_, δ/ppm):8.35–8.33 (d, 4H, Ar, *J* = 8 Hz), 8.17 (s, 2H, Ar), 7.94–7.92 (d, 4H, Ar, *J* = 8 Hz), 7.83–7.79 (t, 6H, triplet, Ar, *J* = 24 Hz), 7.55–7.51 (t, 4 H, Ar, *J* = 16 Hz), 7.41–7.37 (t, 2H, Ar, *J* = 16 Hz), 7.25 (CDCl_3_), 2.9–2.88 (t, 4H, alkyl (-CH_2_-)_2_, *J* = 8 Hz), 1.980 (p, 2H, alkyl -CH_2_-); ^13^C NMR (100 MHz, CDCl_3_, δ/ppm): 152.14, 147.71, 139.45, 137.29, 136.99, 136.35, 135.18, 129.80, 129.45, 128.02, 127.76, 126.86, 124.10, 119.65, 117.80, 31.06, 28.79; HR-MS *m*/*z*: [C_38_H_28_N_6_O_5_] ^+^, 649.2195 (calc. 648.6661).

#### 4.4.5. (2*E*,6*E*)-2,6-*Bis*((3-(4-chlorophenyl)-1-phenyl-1*H*-pyrazol-4-yl)methylene) cyclohexan-1-one **5e**

Light yellow coloured, yield 75%; m.p.130–136 °C; IR (KBr) νmax/cm^−1^: 3055, 2951, 1660, 1596, 1223, 1162, 1060; ^1^H NMR (400 MHz, DMSO- d_6_, δ/ppm): 1.85 (p, 2H, alkyl -CH_2_-), 2.92 (t, 4H, alkyl (-CH_2_-)_2_), 7.37 (t, 2H, Ar, *J* = 12), 7.51–7.63 (m, 14H, Ar), 8.00 (d, 4H, Ar, *J* = 8), 8.83 (s, 2H, Ar); ^13^C NMR (100 MHz, CDCl_3_, δ/ppm): 188.14, 153.65, 139.55, 134.70, 134.47, 130.87, 130.15, 129.68, 129.04, 127.60, 127.38, 127.32, 119.48, 117.38, 28.85, 22.38; HR-MS *m*/*z*: [C_38_H_28_Cl_2_N_4_O] ^+^, 627.1785 (calc. 627.5611).

#### 4.4.6. (2*E*,5*E*)-2,5-*Bis*((1,3-diphenyl-1*H*-pyrazol-4-yl)methylene)cyclopentan-1-one **5f**

Yellow coloured, yield 84%; m.p.250–252 °C; IR (KBr) νmax/cm^−1^: 3056, 2913, 1680, 1600, 1504, 1224, 1171, 1065; ^1^H NMR (400 MHz, DMSO- d_6_, δ/ppm): 3.09 (s, 4H, alkyl (-CH_2_-)_2_), 7.32–7.39 (m, 4H, Ar), 7.47–7.61 (m, 14H, Ar), 8.00 (s, 4H, Ar), 8.85 (s, 2H, Ar); ^13^C NMR (100 MHz, CDCl_3_, δ/ppm): 194.58, 155.12, 139.61, 136.56, 132.17, 129.67, 129.51, 129.08, 128.87, 128.75, 127.67, 127.56, 127.32, 123.94, 119.59, 119.32, 118.19, 26.58; HR-MS *m*/*z*: [C_37_H_28_N_4_O] ^+^, 545.2332 (calc. 544.6444).

#### 4.4.7. (2*E*,5*E*)-2,5-*Bis*((3-(4-methoxyphenyl)-1-phenyl-1*H*-pyrazol-4yl)methylene)cyclopentan-1-one **5g**

Dark yellow coloured, yield 86%; m.p.220–224 °C; IR (KBr) νmax/cm^−1^: 3051, 2912, 1679, 1611, 1530, 1498, 1253, 1174, 1066; ^1^H NMR (400 MHz, CDCl_3_, *δ*/ppm): 3.01 (s, 4H, alkyl (-CH_2_-)_2_), 3.86 (s, 6H, -CH_3_-), 7.01 (d, 4H, Ar, *J* = 4), 7.34 (t, 2H, Ar, *J* = 12), 7.50 (t, 4H, Ar, *J* = 16), 7.62–7.65 (m, 6H, Ar), 7.81 (d, 4H, Ar, *J* = 4), 8.17 (s, 2H, Ar); ^13^C NMR (100 MHz, CDCl_3_, *δ*/ppm): 194.55, 160.05, 154.84, 139.59, 136.39, 130.29, 129.62, 127.44, 127.15, 124.64, 123.97, 119.47, 117.96, 114.29, 55.43, 26.50; HR-MS *m*/*z*: [C_39_H_32_N_4_O_3_] ^+^, 605.2559 (calc. 604.6964).

#### 4.4.8. (2*E*,5*E*)-2,5-*Bis*((1-phenyl-3-(p-tolyl)-1*H*-pyrazol-4-yl)methylene)cyclopentan-1-one **5h**

Light yellow coloured, yield 85%; m.p.234–238 °C; IR (KBr) νmax/cm^−1^: 3062, 2915, 1655, 1584, 1503, 1224, 1173, 1015; ^1^H NMR (400 MHz, CDCl_3_, *δ*/ppm): 2.41 (s, 6H,-CH_3_-), 3.01 (s, 4H, alkyl (-CH_2_-)_2_), 7.29 (d, 2H, Ar, *J* = 8), 7.35 (t, 2H, Ar, *J* = 12), 7.50 (t, 4H, Ar, *J* = 12), 7.59 (d, 4H, Ar, *J* = 4), 7.66 (s, 2H, Ar), 7.81 (d, 4H, Ar, *J* = 12), 8.18 (s, 2H, Ar); ^13^C NMR (100 MHz, CDCl_3_, *δ*/ppm): 194.71, 155.40, 139.92, 138.79, 136.63, 129.81, 129.71, 129.51, 129.12, 127.61, 127.38, 124.24, 119.76, 118.40, 26.79, 21.60; HR-MS *m*/*z*: [C_39_H_32_N_4_O] ^+^, 573.2653 (calc. 572.6976).

#### 4.4.9. (2*E*,5*E*)-2,5-*Bis*((3-(4-nitrophenyl)-1-phenyl-1*H*-pyrazol-4-yl)methylene)cyclopentan-1-one **5i**

Yellow coloured, yield 65%; m.p.>290 °C; IR (KBr) νmax/cm^−1^: 3070, 2933, 1685, 1572, 1534, 1338, 1226, 1168,1090; ^1^H NMR (400 MHz, CDCl_3_, *δ*/ppm): 3.01 (s, 4H, alkyl (-CH_2_-)_2_), 7.37 (t, 2H, Ar, *J* = 16), 7.44–7.53 (m, 8H, Ar), 7.60–7.64 (m, 6H, Ar), 7.80 (d, 4H, *J* = 8), 8.18 (s, 2H); ^13^C NMR (100 MHz, CDCl_3_, *δ*/ppm): 207.17, 194.45, 153.91, 139.54, 136.77, 130.28, 129.73, 129.12, 127.67, 127.51, 123.57, 119.64, 118.14, 31.06, 26.57; HR-MS *m*/*z*: [C_37_H_26_Cl_2_N_4_O] ^+^, 635.2040 (calc. 634.6395).

#### 4.4.10. (2*E*,5*E*)-2,5-*Bis*((3-(4-chlorophenyl)-1-phenyl-1*H*-pyrazol-4-yl)methylene) cyclopentan-1-one **5j**

Yellow coloured, yield 83%; m.p.204–208 °C; IR (KBr) νmax/cm^−1^: 3050, 2911, 1680, 1595, 1500, 1230, 1171, 1065; ^1^H NMR (400 MHz, DMSO- d_6_, *δ*/ppm): 3.08 (s, 4H, alkyl (-CH_2_-)_2_), 7.28 (s, 2H, Ar), 7.40 (t, 4H, Ar, *J* = 8), 7.57 (t, 4H, Ar, *J* = 20), 7.62–7.65 (m, 8H, Ar, *J* = 12), 8.00–8.02 (d, 4H, Ar, *J* = 8), 8.86 (s, 2H, Ar); ^13^C NMR (100 MHz, CDCl_3_, *δ*/ppm): 188.13, 153.92, 139.53, 138.71, 136.76, 130.29, 129.74, 129.13, 127.84, 127.68, 127.52, 125.24, 123.58, 119.64, 118.14, 26.57; HR-MS *m*/*z*: [C_37_H_26_Cl_2_N_4_O]^+^, 613.1554 (calc. 613.5345).

#### 4.4.11. (2*E*,7*E*)-2,7-*Bis*((1,3-diphenyl-1*H*-pyrazol-4-yl)methylene)cycloheptan-1-one **5k**

Light yellow coloured, yield 65%; m.p.210–215 °C; IR (KBr) νmax/cm^−1^: 3052, 2919, 1662, 1597, 1230, 1145, 1061; ^1^H NMR (400 MHz, CDCl_3_, *δ*/ppm): 1.98 (s, 4H, alkyl (-CH_2_-)_4_), 2.75 (s, 4H, alkyl (-CH_2_-)_4_), 7.33 (t, 2H, Ar, *J* = 12), 7.39–7.51 (m, 12H, Ar) 7.72 (d, 4H, Ar, *J* = 8), 7.80 (d, 4H, Ar, *J* = 8), 8.14 (s, 2H, Ar); ^13^C NMR (100 MHz, CDCl_3_, *δ*/ppm): 197.58, 154.54, 139.77, 139.63, 132.46, 129.65, 128.93, 128.81, 128.64, 128.56, 127.08, 126.57, 126.50, 119.47, 117.01, 28.46, 26.66; HR-MS *m*/*z*: [C_39_H_32_N_4_O] ^+^, 573.2656 (calc. 572.6976).

#### 4.4.12. (2*E*,7*E*)-2,7-*Bis*((3-(4-methoxyphenyl)-1-phenyl-1*H*-pyrazol-4-yl)methylene) cycloheptan-1-one **5l**

Light yellow coloured, yield 57%; m.p.115–120 °C; IR (KBr) νmax/cm^−1^: 3070, 2912, 1668, 1591, 1449, 1356, 1249, 1166, 1063; ^1^H NMR (400 MHz, CDCl_3_, *δ*/ppm): 1.75–1.81 (br, s, 4H, alkyl (-CH_2_-)_4,_ *J* = 24), 2.69–2.75 (br, s, 4H, alkyl (-CH_2_-)_4,_ *J* =24), 3.85 (s, 6H, -CH_3_-, *J* = 4), 6.99 (d, 4H, Ar, *J* = 4), 7.32 (t, 2H, Ar), 7.48 (t, 6H, Ar, *J* = 16), 7.64 (d, 4H, Ar, *J* = 4), 7.78 (d, 4H, Ar, *J* = 8), 8.01 (s, 2H, Ar); ^13^C NMR (100 MHz, CDCl_3_, *δ*/ppm): 204.28, 159.92, 153.99, 139.79, 139.44, 129.89, 129.61, 126.91, 126.56, 126.50, 125.07, 119.41, 119.36, 116.46, 114.21, 55.44, 43.52, 31.39, 29.38, 28.59, 25.39; HR-MS *m*/*z*: [C_41_H_36_N_4_O_3_] ^+^, 633.3198 (calc. 632.7495).

#### 4.4.13. (2*E*,7*E*)-2,7-*Bis*((1-phenyl-3-(p-tolyl)-1*H*-pyrazol-4-yl)methylene)cycloheptan-1-one **5m**

Light yellow coloured, yield 63%; m.p.136–141 °C; IR (KBr) νmax/cm^−1^:3062, 2917, 1671, 1592, 1506, 1439, 1339, 1236, 1165, 1067; ^1^H NMR (400 MHz, CDCl_3_, *δ*/ppm): 1.74–1.80 (m, 4H, alkyl (-CH_2_-)_4_), 2.39 (s, 6H, -CH_3_-), 2.68–2.73 (m, 4H, alkyl (-CH_2_-)_4_), 7.26 (d, 4H, Ar, *J* = 12), 7.31 (t, 2H, Ar, *J* = 12), 7.47 (t, 6H, Ar, *J* = 16), 7.59 (d, 4H, Ar, *J* = 8), 7.77 (d, 4H, Ar, *J* = 8), 8.00 (s, 2H, Ar); ^13^C NMR (100 MHz, CDCl_3_, *δ*/ppm): 204.25, 154.25, 139.79, 139.47, 138.36, 129.61, 129.46, 128.52, 126.60, 119.39, 116.64, 43.51, 31.39, 29.37, 28.60, 25.39, 21.47; HR-MS *m*/*z*: [C_41_H_36_N_4_O] ^+^, 601.2966 (calc. 600.7507).

#### 4.4.14. (2*E*,*7E*)-2,7-*Bis*((3-(4-nitrophenyl)-1-phenyl-1*H*-pyrazol-4yl)methylene)cycloheptan-1-one **5n**

Cream coloured, yield 82%; m.p. 132–137 °C; IR (KBr) νmax/cm^−1^: 3051, 2928, 1680, 1597, 1537, 1338, 1226, 1166, 1089; ^1^H NMR (400 MHz, CDCl_3_, *δ*/ppm): 1.75–1.82 (m, 4H, alkyl (-CH_2_-)_4_), 2.65–2.72 (m, 4H, alkyl (-CH_2_-)_4_), 7.33–7.51 (m, 12H, Ar), 7.64 (t, 4H, Ar, *J* = 12), 7.75 (t, 4H, Ar, *J* = 20), 8.00 (s, 2H, Ar); ^13^C NMR (100 MHz, CDCl_3_, *δ*/ppm): 204.15, 197.36, 162.64, 153.22, 152.79, 140.06, 139.62, 130.116, 129.81, 129.67, 129.01, 128.96, 127.19, 126.82, 125.81, 119.48, 119.41, 43.49, 36.60, 31.92, 31.52, 31.35, 29.41, 29.22, 28.57, 28.38, 26.71, 25.40; HR-MS *m*/*z*: [C_39_H_30_N_6_O_5_] ^+^, 663.2353 (calc. 662.6927).

#### 4.4.15. (2*E*,7*E*)-2,7-*Bis*((3-(4-chlorophenyl)-1-phenyl-1*H*-pyrazol-4-yl)methylene)-cycloheptan-1-one **5o**

Canary yellow coloured, yield 37%; m.p. 265–271 °C; IR (KBr) νmax/cm^−1^: 3069, 2938, 1661, 1597, 1227, 1183, 1065; ^1^H NMR (400 MHz, CDCl_3_, *δ*/ppm): 1.98 (s, 4H, alkyl (-CH_2_-)_4_), 2.70–2.75 (m, 4H, alkyl (-CH_2_-)_4_), 7.36–7.41 (m, 4H, Ar), 7.49–7.54 (m, 4H, Ar), 7.78 (t, 4H, Ar, *J* = 20), 7.92 (t, 4H, Ar, *J* = 16), 8.15 (s, 2H, Ar), 8.30–8.35 (m, 4H, Ar); ^13^C NMR (100 MHz, DMSO- d_6_, *δ*/ppm): 203.61, 150.88, 147.65, 141.61, 139.47, 139.21, 130.17, 129.90, 129.55, 129.13, 127.86, 124.62, 124.25, 119.74, 117.11, 43.26, 30.98, 29.42, 28.11, 26.81, 26.29, 25.41; HR-MS *m*/*z*: [C_39_H_30_C_l2_N_4_O] ^+^, 641.1877 (calc. 641.5877).

**Figure 5:**
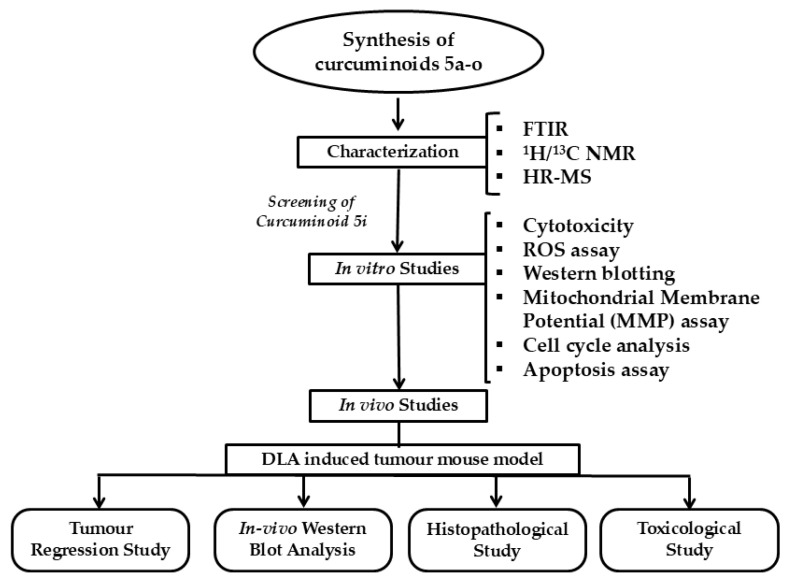
Flowchart summarising experimental approach.

Below in Figure 5, a flowchart summarises the development and evaluation phases of the curcuminoids, providing a clear and concise overview of the experiment approach.

### 4.5. In Vitro Studies

#### 4.5.1. Cell Lines and Cultures

MOLT-4 and HEK-293 cells were acquired from NCCS, Pune. MOLT-4 cells were cultured in RPMI-1640, while HEK-293 cells were grown in DMEM. All media were supplemented with 10% FBS and 1X antimycotic-antibiotic components. Cultures were maintained at 37 °C with 5% CO_2_ in an incubator.

#### 4.5.2. MTT Assay

A total of 10,000 cells per well of MOLT-4 and HEK-293 were seeded in a 96-well plate. The cells were exposed to the specified concentrations of compounds (0.01 µM–10 mM) for 48 h. Following treatment, 5μLof MTT (5 mg/mL) reagent was added to each well and incubated at 37 °C for colour development. MTT a yellow, water-soluble tetrazolium dye gets reduced by mitochondrial dehydrogenases in cells, forming purple formazan granules. The reaction was then halted when formazan colour was developed which was halted by a mix of N, N-Dimethylformamide (50%) and 10% SDS. Cell viability was plotted as a bar graph using the absorbance measured at 570 nm with the SpectraMax i3X microplate reader [47]. IC_50_ of the drugs were calculated using GraphPad Prism (version 8.4.2) using nonlinear regression analysis and dose–responseinhibition method.

#### 4.5.3. Resazurin Assay

MOLT-4 cells were seeded in a 96-well plate at 10,000 cells per well. The cells were treated for 48 h with different drug concentrations ranging from 0.1 µM to 10 mM. After treatment, Resazurin was added to all wells at a final concentration of 22 µM and incubated until the colour changed from blue to pink. Resazurin is a viability dye working on the principle that in living cells, it gets reduced by the respiratory chain in mitochondria to form the non-fluorescent blue resazurin, which produces red fluorescent dye (resorufin). The fluorescence produced was measured using a microplate reader at the excitation wavelength of 550 nm and emission wavelength of 590 nm. Cell viability was represented by a bar graph plotted against the fluorescence produced.

#### 4.5.4. Cell Cycle Analysis

Cell cycle analysis was conducted using flow cytometry. In brief, MOLT-4 cells were seeded at a density of 200,000 cells per well in a 6-well plate and treated with **5i** for 48 h. Following treatment, the cells were harvested, washed with 1X PBS, and fixed in 80% ethanol. After fixation, the cells were collected by centrifugation, washed again with 1X PBS, and incubated overnight with RNase A (50 μg/mL) at 37 °C. The RNase A-treated cell suspension was then stained with 1 μg/mL propidium iodide (PI) and incubated for 30 min in the dark. For each condition, 10,000 events were acquired using a Gallios flow cytometer (Beckman Coulter). The data were analysed and histograms were generated using FCS Express 7 Plus software (De Novo Software).

#### 4.5.5. Apoptosis Assay

The apoptosis assay was conducted using the Annexin V/PI kit (Elabscience, Houston, TX, USA). MOLT-4 cells were seeded at a density of 200,000 cells per well in a 6-well plate and treated with **5i** for 48 h. After incubation, the cells were harvested, washed with 1X PBS, and resuspended in 200 µL of Annexin V binding buffer. Annexin V (5 µL) and PI (5 µL) were added to the cell suspension, followed by a 15min incubation. The samples were then immediately analysed by flow cytometry. For each condition, 10,000 events were acquired using a Gallios flow cytometer (Beckman Coulter, Brea, CA, USA). The data were analysed, and dot plots were generated using FCS Express 7 Plus software (De Novo Software).

#### 4.5.6. JC-1 Mitochondrial Membrane Potential (ΔΨm) Assay

The kit for mitochondrial membrane potential from Elaboscience was utilised. MOLT-4 cells were seeded at 100,000 cells/mL in a 12-well plate and treated with **5i** for 48 h. The cells were collected and resuspended in JC-1 dye after incubating at 37 °C in the dark. After this, the cells were transferred to an ice-cold JC-1 buffer for analysis. Flow cytometry (Gallios, Beckman Coulter) acquired 10,000 events, and a scatter plot illustrated mitochondrial potential using the built-in Galliossoftware version 1.2.

#### 4.5.7. Western Blotting

**5i**-treated and control cells were harvested and lysed in RIPA buffer (1M Tris pH 8, 1% Triton X-100, 0.5% sodium deoxycholate, 1% sodium chloride, 0.1% SDS, 1 mM sodium orthovanadate) supplemented with a protease inhibitor cocktail from MP Biomedicals. The total protein concentration was determined using the Bradford method, and 35 µg of protein was resolved by SDS-PAGE before being transferred to a PVDF membrane. The membrane was cut and probed with the appropriate antibodies. The antibody concentrations were as follows: Bcl-2 (Cloud Clone Corp) at 1:1000, Bax (Cloud Clone Corp) at 1:1000, Bak (CST) at 1:1000, *Cytochrome c* (CST) at 1:500, Cleaved Caspase 3 (CST) at 1:1000, GAPDH (Cloud Clone Corp) at 1:8000, β-actin (Cloud Clone Corp) at 1:8000, Anti-rabbit IgG-HRP (CST) at 1:1000, and Anti-mouse IgG-HRP (CST) at 1:1000. Protein bands were visualised using the enhanced chemiluminescence substrate from Bio-Rad. Images were captured on the Bio-Rad Gel Doc system, and ImageJ 1.52a was used to quantify the protein band intensities.

### 4.6. In Vivo Studies

#### 4.6.1. Animals

Mice were handled and tested according to the guidelines established by the IBAB Animal Ethics Committee and the national regulations of India concerning the care and use of animals. The Institutional Ethics Committee of IBAB, Bangalore, India (Ref. IAEC/IBAB/23/22/7/2023) approved the animal experiments conducted for this study, which also complied with the ARRIVE guidelines. Female Swiss albino mice aged 6–8 weeks (body weight 19–22 g) were obtained from Liveon Biolabs Pvt. Ltd., Bangalore, India. The mice were housed in rooms with controlled humidity, temperature (23 ± 3 °C), and a light cycle (12 h dark/12 h light). Ventilated polypropylene cages accommodated the animals, which were provided with a standard pellet diet from Liveon Biolabs Pvt. Ltd. This standard pellet diet consisted of 21% protein, 5% lipids, 4% crude fibre, 8% ash, 1% calcium, 6% phosphorus, 3.4% glucose, 2% vitamins, and 55% nitrogen-free extract (carbohydrates).

#### 4.6.2. Investigating the Anticancer Potential of **5i** in Mice Models

This study used models of Dalton’s Lymphoma Ascites (DLA). To induce the tumour, 0.5 million cells were injected into the left thigh of the mice. The animals were divided into two groups: The control group *n* = 5 and the treated group *n* = 5. Measurements were recorded on day seven post-tumour implantation using vernier callipers. Group II (treated) animals received intraperitoneal injections of 50 mg/kg body weight of **5i** daily throughout the study period. Preliminary studies determined the dosage. Animal body weights were recorded, and daily measurements of tumour size were taken using vernier callipers. The formula V = (ab^2^)/2 was used to calculate volume, where “a” and “b” represent the major and minor diameters, respectively. Animals were sacrificed at the end of the experimental period, and organs, including tumours, were extracted for staining and protein extraction.

#### 4.6.3. Histological Evaluation of Tumour and Organs (Haematoxylin and Eosin Staining)

The tissues were fixed, processed, and paraffin-embedded following the standard procedure. Sections measuring 5 µm in thickness were cut using a rotary microtome (Leica Biosystems, Wetzlar, Germany) and stained with haematoxylin and eosin as required. The stained sections were examined microscopically, and images were obtained at an appropriate magnification [48].

Statistical analysis: Statistical analysis was conducted using GraphPad Prism version 8.4.2. The significance between the treatment and its paired control was determined using a Student’s *t*-test or one-way ANOVA, followed by Tukey’s multiple comparisons. All data are presented as mean ± standard deviation (SD). The obtained *p*-values were plotted and categorised as * (*p* ≤ 0.05), ** (*p* ≤ 0.01), *** (*p* ≤ 0.001), **** (*p* ≤ 0.0001), and ns as not significant.

## 5. Conclusions

A set of fifteen curcuminoids **5a–o** was prepared, fully characterised, and assessed for cytotoxicity. Of these, we report for the first time a new curcuminoid, (2*E*,5*E*)-2,5-*bis*((3-(4-nitrophenyl)-1-phenyl-1*H*-pyrazol-4-yl)methylene)cyclopentane-1-one **5i**, which demonstrates significantly enhanced anticancer activity against leukemic cells compared to its parent compound, curcumin. Our results reveal that **5i** effectively activates intrinsic apoptosis, which subsequently mediates the inhibition of cell proliferation under both in vitro and in vivo conditions. Moreover, **5i** remarkably inhibits tumour growth with minimal systemic toxicity at low doses, thus making it a promising therapeutic candidate.

## Figures and Tables

**Table 1:** Details of curcuminoids**5a–o**.

Code	R	X
5a	H	-(CH_2_)_3_-
5b	OCH_3_	-(CH_2_)_3_-
5c	CH_3_	-(CH_2_)_3_-
5d	NO_2_	-(CH_2_)_3_-
5e	Cl	-(CH_2_)_3_-
5f	H	-(CH_2_)_2_-
5g	OCH_3_	-(CH_2_)_2_-
5h	CH_3_	-(CH_2_)_2_-
**5i**	NO_2_	-(CH_2_)_2_-
5j	Cl	-(CH_2_)_2_-
5k	H	-(CH_2_)_4_-
5l	OCH_3_	-(CH_2_)_4_-
5m	CH_3_	-(CH_2_)_4_-
5n	NO_2_	-(CH_2_)_4_-
5o	Cl	-(CH_2_)_4_-

**Table 2:** In vitro cytotoxicity data of synthesized curcuminoids **5a-o** on MOLT-4 cell line.

Compound	IC_50_ (µM)
**5a**	34.36 ± 3.24
**5b**	134.59 ± 1.55
**5c**	>100
**5d**	36.39 ± 1.78
**5e**	>100
**5f**	0.92 ± 0.00
**5g**	12.44 ± 0.44
**5h**	49.98 ± 10.77
**5i**	0.10 ± 0.01
**5j**	11.91 ± 1.48
**5k**	0.58 ± 0.00
**5l**	16.15 ± 0.31
**5m**	46.24 ± 4.18
**5n**	8.97 ± 2.62
**5o**	9.23 ± 0.04
Curcumin	61.66 ± 2.26

## Data Availability

All of the relevant data are presented within the paper.

## Abbreviations

The following abbreviations are used in this manuscript:

SAR	Structure–activity relationship
FTIR	Fourier Transform Infrared Spectroscopy
NMR	Nuclear Magnetic Resonance
HR-MS	High-ResolutionMass Spectrometry
MTT	3-(4,5-Dimethylthiazol-2-yl)-2,5-diphenyltetrazolium bromide
IC50	Half-maximal inhibitory concentration
DMSO	Dimethyl sulfoxide
CDCl3	Deuterated chloroform
JC1	5,5′,6,6′-Tetrachloro-1,1′,3,3′-tetraethylbenzimidazolylcarbocyanine chloride
MMP	Mitochondrial membrane potential
DLA	Dalton lymphoma ascites
GF254	SILICA GEL GF 254
TLC	Thin Layer Chromatography
NCCS	National Centre for Cell Science
RPMI	Roswell Park Memorial Institute Medium
FBS	Foetal Bovine Serum
SDS	Sodium dodecyl sulphate
SDS-PAGE	Sodium dodecylsulphate–polyacrylamide gel electrophoresis
PVDF	Polyvinylidene fluoride
ROS	Reactive oxygen species
H&E	Haematoxylin and eosin stain
